# Endophytes and Epiphytes From the Grapevine Leaf Microbiome as Potential Biocontrol Agents Against Phytopathogens

**DOI:** 10.3389/fmicb.2019.02726

**Published:** 2019-11-29

**Authors:** Sébastien Bruisson, Mónica Zufferey, Floriane L’Haridon, Eva Trutmann, Abhishek Anand, Agnès Dutartre, Mout De Vrieze, Laure Weisskopf

**Affiliations:** Department of Biology, University of Fribourg, Fribourg, Switzerland

**Keywords:** endophytes, epiphytes, *Bacillus*, *Botrytis cinerea*, *Phytophthora infestans*, volatiles

## Abstract

Plants harbor diverse microbial communities that colonize both below-ground and above-ground organs. Some bacterial members of these rhizosphere and phyllosphere microbial communities have been shown to contribute to plant defenses against pathogens. In this study, we characterize the pathogen-inhibiting potential of 78 bacterial isolates retrieved from endophytic and epiphytic communities living in the leaves of three grapevine cultivars. We selected two economically relevant pathogens, the fungus *Botrytis cinerea* causing gray mold and the oomycete *Phytophthora infestans*, which we used as a surrogate for *Plasmopara viticola* causing downy mildew. Our results showed that epiphytic isolates were phylogenetically more diverse than endophytic isolates, the latter mostly consisting of *Bacillus* and *Staphylococcus* strains, but that mycelial inhibition of both pathogens through bacterial diffusible metabolites was more widespread among endophytes than among epiphytes. Six closely related *Bacillus* strains induced strong inhibition (>60%) of *Botrytis cinerea* mycelial growth. Among these, five led to significant perturbation in spore germination, ranging from full inhibition to reduction in germination rate and germ tube length. Different types of spore developmental anomalies were observed for different strains, suggesting multiple active compounds with different modes of action on this pathogen. Compared with *B. cinerea*, the oomycete *P. infestans* was inhibited in its mycelial growth by a higher number and more diverse group of isolates, including many *Bacillus* but also *Variovorax, Pantoea, Staphylococcus, Herbaspirillum*, or *Sphingomonas* strains. Beyond mycelial growth, both zoospore and sporangia germination were strongly perturbed upon exposure to cells or cell-free filtrates of selected isolates. Moreover, three strains (all epiphytes) inhibited the pathogen’s growth via the emission of volatile compounds. The comparison of the volatile profiles of two of these active strains with those of two phylogenetically closely related, inactive strains led to the identification of molecules possibly involved in the observed volatile-mediated pathogen growth inhibition, including trimethylpyrazine, dihydrochalcone, and L-dihydroxanthurenic acid. This work demonstrates that grapevine leaves are a rich source of bacterial antagonists with strong inhibition potential against two pathogens of high economical relevance. It further suggests that combining diffusible metabolite-secreting endophytes with volatile-emitting epiphytes might be a promising multi-layer strategy for biological control of above-ground pathogens.

## Introduction

Plants are densely colonized by a variety of microbes (Compant et al., 2019), some of which – the epiphytes – stay on the surface of plant organs, while others are able to penetrate further inside the plants and are called endophytes (Hardoim et al., 2015). Early studies on the structure and function of plant microbiotas have focused on the roots and shown the interplay between soil and host plant in shaping the root and rhizosphere microbiotas (Berg and Smalla, 2009; Philippot et al., 2013). Later investigations demonstrated that the leaves also offer habitats to complex, albeit less diverse microbial communities (Vorholt, 2012). The importance of rhizosphere microbes for plant health is well documented, e.g., through studies on suppressive soils (Mendes et al., 2011; Schlatter et al., 2017), or on the ability of root-colonizing bacteria to stimulate plant defenses, through the so-called “induced systemic resistance” (Pieterse et al., 2014). While the functional roles of phyllosphere inhabitants are still less well understood than those of rhizosphere microbes, few reports have shown that they also significantly contribute to plant health (Innerebner et al., 2011; Ritpitakphong et al., 2016). Indeed, it would seem useful for the plant to be able to count on local defenses provided by phyllosphere microbes to fend off pathogens attacking leaves, in addition to the systemic plant-encoded resistance triggered by root-colonizing microbes. The main aim of this study was therefore to harness the potential of phyllosphere microbiota for protection against above-ground pathogens.

We selected grapevine as a model plant for two main reasons. First, we expected this perennial plant to host a better adapted leaf endophytic microbiome than annual plants, since strong overlap had been reported between communities living in the soil and those colonizing the above-ground plant organs (Zarraonaindia et al., 2015). This suggests that the vineyard soil acts as a reservoir from which microbes colonize the phyllosphere. Second, grapevine cultivation is a heavy consumer of fungicides (Sabatier et al., 2014) due to its high sensitivity to multiple pathogens, among which the oomycete *Plasmopara viticola* causing downy mildew (Gessler et al., 2011) and the ascomycete *Botrytis cinerea* causing gray mold (Pertot et al., 2017). Current disease management practices strongly rely on synthetic fungicides (or copper-based products), but the increasing awareness of the deleterious side-effects of these compounds on environmental and human health urges us to find efficient, less toxic alternative strategies for disease management. Biological control using microbial antagonists of the disease-causing agents represents one such alternative strategy (Pal and Mc Spadden Gardener, 2006; Syed Ab Rahman et al., 2018). In grapevine, the search for biocontrol agents has been ongoing for some years to find alternative solutions against gray mold as well as against downy mildew. Several fungal and bacterial biological control agents are available for the control of gray mold (recently reviewed in Abbey et al., 2019), while only few have been reported for downy mildew (Dagostin et al., 2011; Puopolo et al., 2014b). In contrast to synthetic fungicides, biological control agents offer the advantage of having various modes of action (Haidar et al., 2016), which can be direct (e.g., through antibiosis or niche competition) or indirect, through induction of resistance (Pertot et al., 2016). The latter has been object of many studies dealing with grapevine protection against diseases (Delaunois et al., 2014; Aziz et al., 2016).

Independently of the mode of action of biological control agents, one major difficulty associated with their use is their inconsistent performances in the field. Combination of few biological control agents has been suggested as a mean to increase robustness by providing functional redundancy as well as complementarity in the modes of action (De Vrieze et al., 2018), yet some studies have shown that single strains can perform as well as mixed consortia (Pertot et al., 2017). Moreover, most commercially available biocontrol strains have not been isolated from the plant/organ they are intended to protect, which might at least partially account for their inconsistent survival and efficacy in the field. Moreover, most available bacterial biocontrol agents originate from the rhizosphere but are used as leaf sprays, although both plant habitats greatly differ, as do their native microbial colonizers (Müller et al., 2016). For this reason, the major aim of this study was to isolate bacteria from grapevine leaves and to test their potential suitability for the biological control of important disease-causing agents. Different cultivars differ in their sensitivity to diseases, but the relative contribution of the host plants and their microbiotas in mounting this resistance has not yet been elucidated. We therefore compared isolates obtained from two sensitive varieties, Pinot Noir and Chasselas, with those from the disease-resistant variety Solaris. Moreover, we isolated both epiphytic and endophytic bacteria in order to discriminate between tightly associated bacteria and those more loosely connected to the leaves. After taxonomic identification, we assembled a collection of 78 non-redundant strains, which we tested for *in vitro* growth inhibition of *Botrytis cinerea* and *Phytophthora infestans.* The latter was used as a surrogate oomycete pathogen for *Plasmopara viticola*, which cannot be cultivated *in vitro.* In addition to classical dual assays, we also tested the potential of the grapevine isolates to inhibit the growth of phytopathogens through the emission of volatile compounds, which have gained attention in recent years as promising antifungal agents emitted by plant-associated bacteria (Bailly and Weisskopf, 2017). Finally, we mined our grapevine leaf strain collection for phylogenetically closely related strains of differing volatile-mediated activity to identify candidate molecules underlying the observed pathogen-inhibiting effects.

## Materials and Methods

### Microbial Strains and Culture Media

In total, 194 bacteria were isolated as described earlier (Vionnet et al., 2018) from both the epiphytic and endophytic compartments of three grapevine cultivars, one resistant to fungal diseases (Solaris) and two sensitive to fungal diseases (Pinot noir and Chasselas). Bacterial strains were routinely grown on LB (Luria-Bertani) medium and incubated in the dark at room temperature. LB medium was prepared by dissolving 12.5 g/L of LB Broth Miller and 10 g/L of LB Broth Lennox (Fisher Bioreagents)^1^ in distilled water, to which 15 g/L of Agar-Agar Kobe I (Roth)^2^ were added for solid LB medium. *Botrytis cinerea* strain BMM was provided by Brigitte Mauch-Mani (University of Neuchâtel, Switzerland) and grown on Potato dextrose Agar (PDA). This medium was prepared by dissolving 39 g of PDA Powder (Sigma-Aldrich) in one liter of distilled water. *Botrytis cinerea* plates were incubated at 20°C with 12 h light/12 h dark cycle. *Phytophthora infestans* strain Rec01 (Hunziker et al., 2015) was grown on V8 agar medium and incubated at 18°C in the dark. V8 100% hot spicy vegetable juice^3^ was prepared at 100 mL/L in distilled water and 1 g/L of CaCO_3_ was added. Agar (15 g/L) was added for solidified V8. Each medium was sterilized by autoclaving at 120°C during 20 min. For long-term storage, bacterial strains were kept at -80°C in 25% glycerol (Reactolab SA) in cryogenic tubes (Sarstedt). *B. cinerea* and *P. infestans* were kept as mycelial plugs in 10% glycerol in a nitrogen tank after a few hours at −20°C and after one night at −80°C.

### Taxonomic Identification of Grapevine Epiphytes and Endophytes

For taxonomic identification, amplification of the full-length 16S rRNA gene was carried out. To this end, two to three colonies of each bacterial strain were lysed in 50 μL of distilled water by boiling at 100°C during 10 min. The polymerase chain reaction (PCR) was performed in a total volume of 25 μL with 5 μL of lysed bacterial solution as template. The primers BactF (5′-AGA GTT TGA TYM TGG CTC-3′) and BactR (5′-CAK AAA GGA GGT GAT CC-3′) were used at a final concentration of 0.5 μM, while the loading gel track and the polymerase Accustart II PCR toughmix (VWR) were added according to the manufacturer’s protocol. The PCR program was performed as follows: an initial denaturation step at 94°C for 3 min, then 35 cycles of three steps made up of a denaturation step at 94°C during 30 s, an annealing step at 56°C during 30 s and an extension step at 72°C during 1.5 min, followed by a final extension step at 72°C during 10 min. Three μL of PCR product were run on a 1% agarose gel to verify the correct size of the amplified product. The rest of the PCR product was purified by QIAquick PCR purification Kit (Qiagen) following the protocol of the manufacturer. Twelve μL of sample were mixed with 3 μL of either the BactF or the BactR primer at 10 μM and were sent to an external company for sequencing (Microsynth). The obtained chromatograms were visually inspected and sequences were manually corrected when necessary.

### Assembly of a Collection of Non-redundant Strains and Construction of a Phylogenetic Tree

In order to identify redundant isolates (=same bacterial strain isolated twice), all sequences retrieved from bacteria isolated from the same cultivar and compartment (endo- vs. epiphytes) were aligned using the Geneious software. In each of these subsets of sequences, strains having a distance matrix inferior to 0.2 were considered redundant and only one representative of these redundant strains was selected for further analysis. In total, 78 strains were kept in the collection of non-redundant strains. These sequences have been submitted to the NCBI database (accession numbers MN555571-MN555648). The 78 sequences were then blasted using the nucleotide database from NCBI^4^ to determine the genus and species affiliation for each strain. The blast results are listed in the supplement (Supplementary Table S1). A phylogenetic tree based on the sequences of these 78 non-redundant isolates was constructed using the following parameters in the Geneious software: a global alignment with free end gaps was performed, with a similarity index of 65%. A neighbor-joining tree was then constructed using the Tamura-Nei genetic distance model. In addition to the grapevine isolates, sequences corresponding to known bacterial species of the following genera were added prior to the alignment: *Variovorax* (NR_113736.1), *Cupriavidus* (NR_074704.1), *Erwinia* (NR_148650.1), *Sphingomonas* (NR_104893.1), *Rhodopseudomonas* (NR_114302.1), *Methylobacterium* (NR_115219.1), *Microbacterium* (NR_042480.1), *Micrococcus* (NR_134088.1), *Paenibacillus* (NR_044524.1), and *Sporosarcina* (NR_025049.1). For the *Bacillus* and *Staphylococcus* genera, the sequences from the following species were added: *B. circulans* (FJ581445.1), *B. aryabhattai* (NR_115953.1), *B. butanolivorans* (MN235850.1), *B. zhangzhouensis* (NR_148786.1), *B. stratosphericus* (MH973204.1), *B. licheniformis* (NR_118996.1), *B. subtilis* (NR_113265.1), *B. halotolerans* (NR_115063.1), *B. cereus* (NR_074540.1), *S. saprophyticus* (L37596.1), *S. hominis* (L37601.1), *S. epidermidis* (NR_113957.1), *S. pasteuri* (NR_114435.1) and *S. warneri* (NR_025922.1). A *Flavobacterium* sequence (MK246909.1) was used as an outgroup.

### *In vitro* Dual Assays

The antagonistic activity of the 78 non-redundant isolates was tested against both pathogens (*B. cinerea* and *P. infestans*) on standard (Greiner bio-one) and (Sarstedt) Petri dishes. V8 medium was used for standard plates and a combination of V8 (fungus/oomycete) and LB (bacteria) media were used for two-compartment plates. The assays on standard plates allowed both volatile and diffusible compounds to be exchanged between the two partners, while only volatiles could be exchanged in the split plate assays (Supplementary Figure S1). Liquid cultures of the bacterial strains were prepared by suspending two to three colonies from fresh LB agar cultures in 3–5 mL of sterile LB broth and incubating overnight at 28°C under shaking at 180 rpm. After a first centrifugation step at 5000 rpm during 5 min, the supernatant was removed. The bacterial cell pellet was washed one time in 0.9% (w:v) NaCl with the same centrifugation conditions and the bacterial pellet was then resuspended in 0.9% NaCl. Optical density (OD) was measured at 595 nm to evaluate cell density, and was adjusted to 1 with 0.9% NaCl. Three drops of 10 μL of OD_595_ = 1 bacterial suspension were then inoculated at the border of standard Petri dishes, and at the border of one compartment for the split Petri dishes (Supplementary Figure S1). The pathogens (*B. cinerea* and *P. infestans*) were inoculated by placing a 5 mm plug of a 3-day-old *B. cinerea* culture grown on PDA medium plate or of a 2-week-old *P. infestans* culture grown on V8 agar medium plate at the center of the standard Petri dishes and at the center of one compartment of the split Petri dishes (Supplementary Figure S1). Plates were then sealed with Parafilm M. For dual assays with *P. infestans*, both partners (bacterium and oomycete) were inoculated on the same day. For dual assays with *B. cinerea*, the bacteria were given a head start of 3 days before fungus inoculation to take into account the very fast growth of the pathogen. Control plates without bacteria were supplemented with three droplets of 10 μL of 0.9% NaCl and one plug of the respective pathogen mycelium. Dual assay plates were incubated in the dark at 23°C and pictures were taken at several time points during incubation. Pictures corresponding to the time point when *B. cinerea* and *P. infestans* reached the border of the Petri dishes in the control plates were used to measure mycelial growth with ImageJ and to calculate the percentage of growth inhibition caused by bacterial exposure. For dual assays on full Petri dishes, the inhibition area (*A*_i_) was calculated as follow: *A*_i_ = *A*_c_ - *A*_b_ - *A*_t_, where *A*_c_ is the mean of the area colonized by the pathogen in the control plates, *A*_b_ is the area colonized by the bacteria in the test plate and *A*_t_ is the area colonized by the pathogen in the test plate. The percentage of growth inhibition was given by the formula: inhibition percentage = 100 × *A*_i_/(*A*_c_ – *A*_b_), where *A*_c_ – *A*_b_ is the area available for the pathogen growth in test plates (Supplementary Figure S2a). For dual assays on two-compartment Petri dishes, the inhibition area was calculated as: *A*_i_ = *A*_c_ - *A*_t_ and the percentage of growth inhibition was calculated as: 100 × *A*_i_/*A*_c_ (Supplementary Figure S2b). Statistical analysis was performed using a two-tailed Student’s *t*-test, ^∗^*p* < 0.05, ^∗∗^*p* < 0.01, and ^∗∗∗^*p* < 0.001.

### Effects of Selected Bacterial Strains on *B. cinerea* Spore Germination

The spores of *B. cinerea* were harvested from a PDA culture in sterile water and filtered through glass wool to remove hyphae. After a centrifugation step at 800 rpm for 10 min, the spore pellet was suspended in 100 μL of sterile water. The spore concentration was determined and diluted at 2 × 10^5^ spores/mL in clarified V8 medium [i.e., medium filtered through a PVDF 0.2 μm syringe filter (Fisher Scientific)]. Six *Bacillus* strains were selected according to their inhibitory effect on the mycelial growth of *B. cinerea*: CHD4, CHP14, PID5, SOD5, SOD20, and SOP51. Moreover, two strains showing no inhibitory effect on the mycelium growth of *B. cinerea* were chosen as controls (PIP26, SOP5). The effect of the bacterial strains on the germination of *B. cinerea* spores was tested using two modalities, one with the bacterial cells and one with their spent medium (cell-free filtrate). Two to three bacterial colonies grown on LB agar medium were inoculated in filtered V8 broth and incubated overnight at 28°C under 180 rpm shaking. The overnight bacterial liquid culture was diluted to OD_595_ = 1 in filtered V8 broth. To obtain cell-free filtrates, bacterial cultures with OD_595_ = 1 were filtered with sterile syringe filters PVDF 0.2 μm (Fisher Scientific). Nine μL of bacterial culture or filtered bacterial culture were mixed with 3 μL of *B. cinerea* spores at a final concentration of 5 × 10^4^ spores/mL on a glass slide. Controls contained 9 μl of filtered V8 medium instead of bacterial culture or cell-free filtrate. The glass slides were placed in a humid box and incubated for 8 and 24 h, respectively. The germination of spores was observed using a Leica DMR microscope with bright-field settings. At least 30 spores of *B. cinerea* were analyzed. The analysis criteria were the percentage of germinated spores, the percentage of spores with multiple germ tubes as well as the percentage and type of anomalies observed. Three different anomalies were distinguished: hyphal swelling, swelling at the germ tube tip and multiple swellings in a row. In addition, germ tube length was measured using ImageJ. All above-mentioned parameters were scored after 8 h of incubation. Statistical analysis was performed using a Chi-square test with Yate’s correction for continuity (*p* < 0.05) to reveal differences in percentages of germination and in percentages of abnormal germination between the bacterial treatments and the control. For the germ tube lengths, a two-tailed Student’s *t*-test was used (^∗^*p* < 0.05, ^∗∗^*p* < 0.01, and ^∗∗∗^*p* < 0.001). Furthermore, the presence of bacterial accumulation around spores, the occurrence of spore or germ tube vacuolization and the production of conidiophores were assessed at a later time point (24 h after inoculation).

### Effects of Selected Bacterial Strains on *P. infestans* Zoospore and Sporangia Germination

Zoospores of *P. infestans* were collected by adding 10 mL of ice-cold sterile water to 12–14 days old plates of *P. infestans* grown on V8 medium. The plates were incubated for 2 h at 4°C. After incubation, the plates were left at room temperature in the dark for 20 min. Swimming zoospores were collected by pipetting the upper layer of liquid from the Petri dishes. Zoospore concentration was 1.8 × 10^5^ spores/mL. Sporangia of *P. infestans* were harvested by scraping off the mycelium of 12–14 days old cultures grown on V8 medium and by suspending the mycelium in sterile water. After vigorous shaking, the suspension was filtered using a mesh to remove the hyphae. Sporangia concentration was adjusted to 2 × 10^5^ spores/mL. As with *B. cinerea*, bacterial isolates CHD4, CHP14, PID5, SOD5, SOD20, and SOP51 showed strong inhibitory potential on the mycelium growth of *P. infestans* and were therefore selected, with non-active bacterial strains PIP26 and SOD5 added as controls. Bacterial strains were prepared as described above for the germination of *B. cinerea* spores and the effects on zoospores and sporangia germination of both the bacterial cells and their spent medium (cell-free filtrate) were assessed. Nine μL of bacterial culture or filtered bacterial culture were mixed with 3 μL of zoospores or sporangia suspensions at a final concentration of 4.5 × 10^4^ and 5 × 10^4^ spores/mL respectively. Controls contained 9 μl of filtered V8 medium instead of bacterial culture or cell-free filtrate. For the zoospores, the mixtures were pipetted on the inner side of the lid of 96-well plates, in order to provide a solid surface for the zoospores to adhere to prior to germination. For the sporangia, the mixtures were pipetted onto water agar disks (0.8%, Ø 8 mm) in Petri dishes. The 96-well plates and Petri dishes were placed in a humid box and incubated at 18°C for 4 and 24 h respectively. Germination of both type of spores was observed using a Leica DMR microscope with bright-field settings. Pictures of the mixtures of bacteria with zoospores and sporangia were taken at a 10-fold and 5-fold magnification respectively and analyzed. Counts of germinated and non-germinated zoospores were performed. On average, the pictures contained 60 zoospores or 75 sporangia. Additionally, for the germinating zoospores, normal, delayed and abnormal germination of zoospores were distinguished in the counting. Percentage of germination and the percentages of the different germinated zoospore phenotypes were computed. Differences between bacterial treatments and the control were assessed by using a two-tailed Student’s *t*-test (^∗^*p* < 0.05, ^∗∗^*p* < 0.01, and ^∗∗∗^*p* < 0.001) for zoospore and sporangia germination. A Chi-square test with Yate’s correction for continuity (*p* < 0.05) was performed to compare the detailed germination behavior of the zoospores.

### Collection and Analysis of Volatiles Emitted by Selected Strains

Two strains exhibiting volatile-mediated inhibition of *P. infestans* were selected for volatile analysis: CHP14 and CHP20. Furthermore, two closely related strains with no volatile-mediated activity were selected for comparison: CHD4 (closely related to CHP14) and SOD6 (closely related to CHP20). For each of these four strains, two or three colonies from a LB agar plate were resuspended in 3 mL of LB broth, incubated 24 h at 28°C and shaken at 190 rpm. The bacterial culture was adjusted to a density of OD_595_ = 1.0 in LB broth and 100 μL of this cell suspension were spread on LB agar medium poured into 5 cm glass Petri dishes. LB broth inoculated on LB-agar glass plates was used as control. The strains were grown overnight at room temperature before collecting the volatiles during 48h using closed-loop-stripping analysis (CLSA) as described by Hunziker et al. (2015). Trapped volatiles were extracted from the charcoal filter by rinsing the filter three times with 25 μL dichloromethane (≥99.8%, VWR). The experiment was repeated four times. The volatiles were analyzed by gas chromatography-mass spectrometry (GC/MS). The samples were injected in a HP6890 gas chromatography connected to a HP5973 mass selective detector fitted with an HP-5 ms fused silica capillary column (30 m; 0.25-mm inside diameter; 0.25–μm film; Agilent Technologies). Conditions were as follows: inlet pressure, 67 kPa; He, 15 mL/min; injection volume, 2 μL; transfer line, 300°C; injector, 250°C; electron energy, 70 eV. The gas chromatograph was programed as follows: 5 min at 50°C, then increasing 5°C/min to 320°C and hold for 1 min. MZmine-2.20 was used to process the chromatograms. Statistical analysis of the processed data was performed using Metaboanalyst 3.0 (Xia et al., 2015). The differentially enriched compounds were identified using NIST 17 database using OpenChrom software.

## Results

### Diversity of Cultivable Bacteria in Grapevine Leaves

**FIGURE 1:**
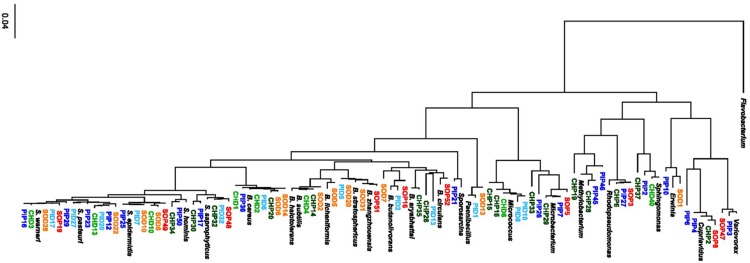
Phylogenetic tree of 78 non-redundant isolates from the microbiome of grapevine leaves. The tree was constructed using the Genious software (see Materials and Methods for details). Isolates from Pinot Noir are shown in blue, with epiphytes (PIP) in dark blue and endophytes (PID) in light blue. Isolates from Solaris are shown in red/orange, with epiphytes (SOP) in red and endophytes (SOD) in orange. Isolates from Chasselas are shown in green, with epiphytes (CHP) in dark green and endophytes (CHD) in light green. A *Flavobacterium* sequence (MK246909.1) was used as an outgroup and reference sequences from different genera and different species within the *Bacillus* (B.) and *Staphylococcus* (S.) genera corresponding to the following accession numbers were included: *Variovorax* (NR_113736.1), *Cupriavidus* (NR_074704.1), *Erwinia* (NR_148650.1), *Sphingomonas* (NR_104893.1), *Rhodopseudomonas* (NR_114302.1), *Methylobacterium* (NR_115219.1), *Microbacterium* (NR_042480.1), *Micrococcus* (NR_134088.1), *Paenibacillus* (NR_044524.1), *Sporosarcina* (NR_025049.1), *B. circulans* (FJ581445.1), *B. aryabhattai* (NR_115953.1), *B. butanolivorans* (MN235850.1), *B. zhangzhouensis* (NR_148786.1), *B. stratosphericus* (MH973204.1), *B. licheniformis* (NR_118996.1), *B. subtilis* (NR_113265.1), *B. halotolerans* (NR_115063.1), *B. cereus* (NR_074540.1), *S. saprophyticus* (L37596.1), *S. hominis* (L37601.1), *S. epidermidis* (NR_113957.1), *S. pasteuri* (NR_114435.1), and *S. warneri* (NR_025922.1).

**FIGURE 2:**
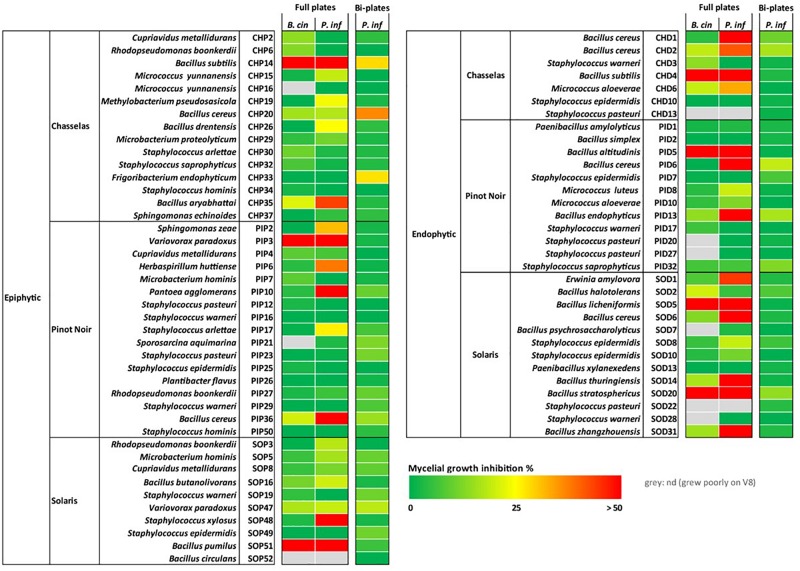
Inhibiting activity of grapevine bacterial isolates on the mycelial growth of two phytopathogens. Strains were tested in standard (full) plates and two-compartment plates for *in vitro* inhibition of mycelial growth of *Botrytis cinerea* (*B. cin*) and of *Phytophthora infestans* (*P. inf*). Pathogens and bacteria were grown on V8 medium in both setups. Bacteria were grown on V8 for full plate assays and on LB for split plate assays. No inhibition of *B. cinerea* was observed using the two-compartment plate setup, therefore only data for *P. infestans* are shown. This experiment was performed with 3–5 replicates for each strain and a color code is shown with red indicating highest inhibition and green no inhibition or promotion of growth. The corresponding numerical data and statistical analysis are presented in Supplementary Table S2. Four strains depicted in Figure 1 are not shown here because they grew poorly on V8 and on LB: CHD40, CHP28, PIP45, and PIP46. Species names of the strains correspond to the closest blast hits (Supplementary Table S1).

The 16S rRNA sequencing of the 194 bacterial strains isolated from grapevine leaf wash off (= epiphytes) and from surface-sterilized leaves (= endophytes) revealed 78 non-redundant strains. A large majority of these were Gram-positive bacteria, with a strong representation of two genera: *Bacillus* and *Staphylococcus.* As expected, the diversity on genus level was much lower among endophytes than among epiphytes (Figures 1, 2): except for three *Micrococcus*, two *Paenibacillus* strains and one *Erwinia* strain, most endophytes belonged to the *Bacillus* or *Staphylococcus* genera. The proportions between these two genera among endophytes differed in the three cultivars: they were similarly frequent (38%) in Chasselas, while *Staphylococcus* were slightly more frequently isolated (42%) than *Bacillus* (33%) in Pinot Noir (Supplementary Table S1). In the disease-resistant Solaris cultivar, *Bacillus* isolates constituted 54% of total endophytes, while *Staphylococcus* represented 31%. Overall, only two Gram-negative isolates (one *Erwinia* and one *Sphingomonas*) were found among endophytes in our survey (Supplementary Table S1). Gram-negative bacteria were more frequently retrieved among epiphytes, including *Cupriavidus*, *Methylobacterium*, or *Rhodopseudomonas* strains (Figure 1). In addition to *Bacillus* and *Staphylococcus* strains, which were also isolated from epiphytic communities of all three cultivars, nine different genera were found among Pinot Noir epiphytes, seven among Chasselas and four among Solaris epiphytes. *Bacillus* isolates made up one third of the isolated epiphytes in Chasselas and Solaris, but only 5% of Pinot Noir epiphytes (Figure 1 and Supplementary Table S1).

### Screening for Antagonistic Activity of Grapevine Leaf Isolates Against Pathogens

In dual assays allowing exchange of both volatile and diffusible metabolites (full plates), mycelial growth inhibition of *P. infestans* was more widespread than the inhibition of *B. cinerea* for both epiphytes and endophytes. With one exception (*Variovorax* strain PIP3), only *Bacillus* strains were able to strongly reduce mycelial growth in *B. cinerea*, while significant inhibition of *P. infestans* was also observed for strains belonging to the genera *Pantoea, Variovorax, Staphylococcus, Erwinia, Sphingomonas, Herbaspirillum* or *Micrococcus* (Figure 2 and Supplementary Table S2). The ability to inhibit the mycelial growth of *P. infestans* was more frequently observed among endophytes (41%) than among epiphytes (21%). This tendency was also visible for *B. cinerea*, although less pronounced (Figure 2). When only volatile compounds were allowed to reach the target pathogens (two-compartment plates), the strains did not inhibit the growth of *B. cinerea.* However, *P. infestans* was significantly inhibited in its growth by a few strains, which were all epiphytes from Chasselas (Figure 2 and Supplementary Table S2). Two of these emitters of growth-inhibiting volatiles belonged to the *Bacillus genus*: one clustered with the *B. subtilis* group (CHP14), and the second with the *B. cereus* group (CHP20). The third strain was also a Gram-positive bacterium belonging to the genus *Frigoribacterium* (CHP33) (Figure 1). Moderate *P. infestans* growth inhibition also occurred upon exposure to volatiles from endophytes, but was more frequently observed among epiphytes (Figure 2 and Supplementary Table S2). It should be noted that in this two-compartment plate bioassay, bacteria were grown on LB medium, not on V8, since the volatiles emitted when the bacteria grew on V8 did not lead to significant pathogen growth reduction (data not shown).

### Selected *Bacillus* Isolates Strongly Affected Spore Germination of *Botrytis cinerea*

**FIGURE 3:**
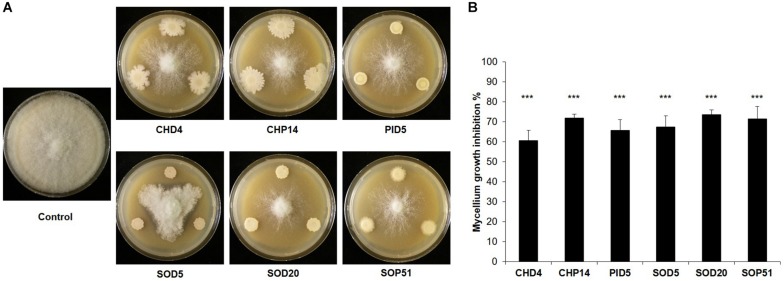
Inhibition of mycelial growth of *Botrytis cinerea.* Representative pictures of dual assays with *Botrytis cinerea* non-exposed to the bacteria (Control) or exposed to six bacterial strains isolated from the grapevine microbiome are shown in **(A)**. Pictures were taken 6 days after *B. cinerea* inoculation. The percentage of *B. cinerea* mycelium inhibition induced by each strain compared to the non-exposed control that represents 100% is shown in **(B)**. Bars represent average inhibitions of five replicates with standard deviation. The percentage of inhibition was determined 6 days after *B. cinerea* inoculation. Asterisks represent statistically significant differences in comparison to the control (*t*-test; *p* < 0.001). The experiment was repeated twice with similar results.

**FIGURE 4:**
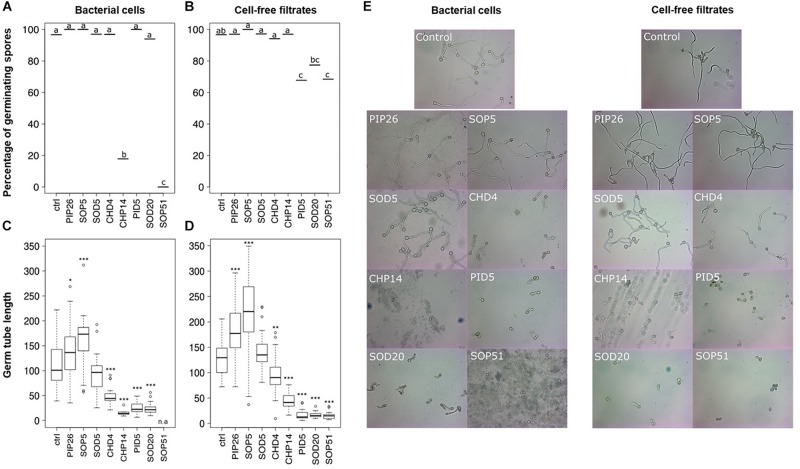
Inhibition of spore germination of *Botrytis cinerea.* Percentages of germinated spores observed (out of ca. 30 spores) after 8h upon exposure to bacterial cells are shown in **(A)**, those observed upon exposure to cell-free filtrates are shown in **(B)**. Different letters indicate statistically significant differences according to a Chi-square test with Yate’s correction for continuity (*p* < 0.05). The length of the germ tube was measured with ImageJ in germinated spores exposed to bacterial cells (*n* = 5–39) **(C)** or cell-free filtrates (*n* = 21–34) **(D)**. Stars indicate significant difference in germ tube length compared with the respective control (ctrl) according to a *t*-test (^∗^*p* < 0.05; ^∗∗^*p* < 0.01; ^∗∗∗^*p* < 0.001). n.a., not available (no spore germinated in this treatment). Representative pictures of spores co-incubated with bacterial cells (left) or cell-free filtrates (right) are shown in **(E)**. The experiment was repeated twice with similar results.

**FIGURE 5:**
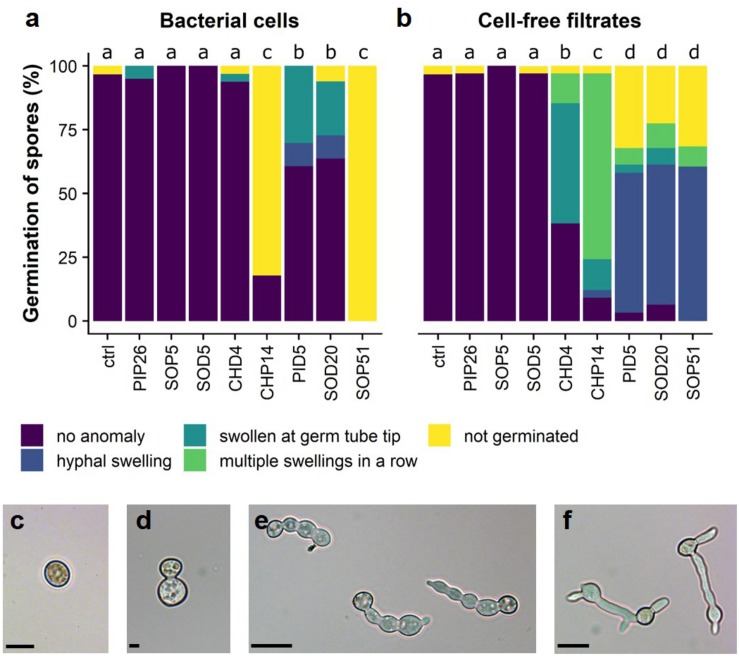
Perturbed development of germinated spores of *B. cinerea.* Proportions of anomalies observed in bacterial cell-exposed spores are shown in **(a)**, those observed in cell-free filtrate-exposed spores are shown in **(b)**. Different letters indicate significantly different proportions of anomalies according to a Chi-square test with Yate’s correction for continuity (*p* < 0.05). The different types of anomalies observed in germinating spores of *B. cinerea* are shown in **(c–f)***:* **(c)** not germinated; **(d)** hyphal swelling; **(e)** multiple swellings in a row; **(f)** swelling at germ tube tip. Scale bars = 30 μm. The experiment was repeated twice with similar results.

The above-mentioned screen for antagonistic activity revealed six *Bacillus* isolates that reduced mycelial growth of *B. cinerea* by 57–74% (Figure 2 and Supplementary Table S2). The dual assay was repeated for these six strains and led to the same results, which are shown with representative pictures in Figure 3. Interestingly, these six *Bacillus* strains (CHP14, CHD4, SOP51, PID5, SOD5, and SOD20) were phylogenetically closely related and clustered together in a particular subgroup of the tree with reference strains such as *B. subtilis, B. zhangzhouensis, B. licheniformis*, or *B. stratosphericus* (Figure 1). To characterize the antifungal potential of these strains beyond mycelial growth inhibition, their ability to interfere with spore germination was assessed by co-inoculating *B. cinerea* spores with either the bacterial cells themselves or their spent medium (cell-free filtrate). Two strains showing no activity on mycelial growth (PIP26 and SOP5) were included as negative control in this analysis. In contrast to their effect on mycelial growth, the six *Bacillus* strains strongly differed in their impact on spore germination when applied as cells (Figures 4A,E): SOD5, CHD4, PID5, and SOD20 had no or only very marginal effect on the pathogen’s germination rate, while CHP14 reduced it to 20% and SOP51 to 0%. When applied as cell-free filtrates, the effects of the strains on germination rate were generally less strong, although PID5, SOP51, and to a lesser extent SOD20, still caused marked reductions (Figures 4B,E). When measuring the length of the germ tube, significant reduction was obtained with all except SOD5 with both cells (Figure 4C) and cell-free filtrates (Figure 4D). Not only did the strains cause drastic tube length reduction, but they also led to specific developmental anomalies (depicted in Figure 5) such as hyphal swelling, multiple swelling in a row or swelling at the end of the germ tube. Proportions of these different phenotypes were strain specific and differed between spores exposed to cells and those exposed to cell-free filtrates of the same isolate (Figures 5a,b). For instance, multiple swelling in a row only occurred when exposed to cell-free filtrates and hyphal swelling was much more frequently observed in filtrate-treated than in cell-treated spores (Figures 5a,b), which might be partially due to the lower proportion of non-germinating spores in filtrate-treated compared with cell-treated samples (Figure 5). In order to see whether the active strains (or their filtrates) only delayed germination or completely arrested it, spores were analyzed for germination rate, conidiophore production, the above-described anomalies, as well as vacuolization and degradation after 24 h of incubation. The results are summarized in Table 1 and illustrated in Supplementary Figure S3. Spore germination was observed for all treatments after 24 h (Table 1), but it was severely reduced in samples exposed to cells and cell-free filtrates of all strains but the inactive strains PIP26, SOP5 as well as SOD5, which confirmed the earlier time point observations (Table 1 and Supplementary Figure S3). CHD4 had a strong inhibitory effect on germination when applied as cell suspension but its filtrate did not prevent the formation of conidiophores nor did it lead to vacuolization or visible degradation of fungal structures, unlike all other active strains (Table 1 and Supplementary Figure S3).

**TABLE 1:** Presence or absence of different phenotypes in spores exposed to bacterial cells or their spent medium (filtrate) for 24 h.

	**Germination**		**Conidiophores**		**Anomalies**		**Vacuolization**		**Degradation**	
					
	**Cells**	**Filtrate**	**Cells**	**Filtrate**	**Cells**	**Filtrate**	**Cells**	**Filtrate**	**Cells**	**Filtrate**
Control	Yes	Yes	Yes	Yes	No	No	No	No	No	No
PIP26	Yes	Yes	Yes	Yes	No	No	No	No	No	No
SOP5	Yes	Yes	Yes	Yes	No	No	No	No	No	No
SOD5	Yes	Yes	Yes	Yes	No	No	No	No	No	No
CHD4	Yes	Yes	No	Yes	Yes	Yes	No	No	Yes	No
CHP14	Yes	Yes	No	No	Yes	Yes	Yes	Yes	Yes	Yes
PID5	Yes	Yes	No	No	Yes	Yes	Yes	Yes	Yes	Yes
SOD20	Yes	Yes	No	No	Yes	Yes	Yes	Yes	Yes	Yes
SOP51	Yes	Yes	No	No	Yes	Yes	Yes	Yes	Yes	No

*Ten pictures per treatment were examined for the presence of the described phenotypes.*

### Both Zoospores and Sporangia From *Phytophthora infestans* Were Affected in Their Germination by the Selected *Bacillus* Strains

**FIGURE 6:**
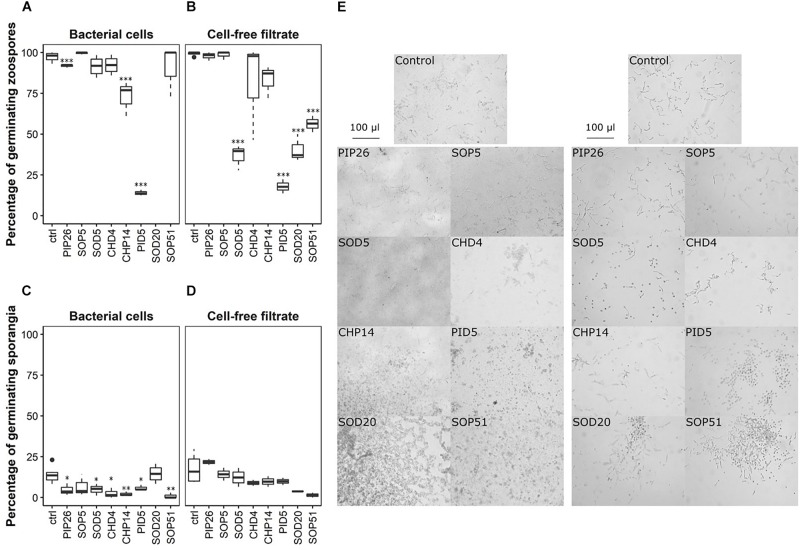
Inhibition of zoospore and sporangia germination of *P. infestans.* Percentages of germinated zoospores observed after 4 h upon exposure to bacterial cells are shown in **(A)**, those observed upon exposure to cell-free filtrates are shown in **(B)**. Percentages of germinated sporangia after 24 h upon exposure to bacterial cells and to cell-free filtrates are shown in **(C,D)** respectively. Stars indicate significant difference in germination percentage compared with the respective control (ctrl) according to a *t*-test (^∗^*p* < 0.05; ^∗∗^*p* < 0.01; ^∗∗∗^*p* < 0.001). Both experiments were replicated once, generating two datasets of which one is represented in this figure, and three technical replicates were prepared, except for the effect of the cell-free filtrates on sporangia germination, for which only two technical replicates were analyzed. Per technical replicate, one picture was taken and analyzed. Representative pictures of zoospores co-incubated with bacterial cells (left) or cell-free filtrates (right) are shown in **(E)**. The experiment was repeated twice with similar results.

**FIGURE 7:**
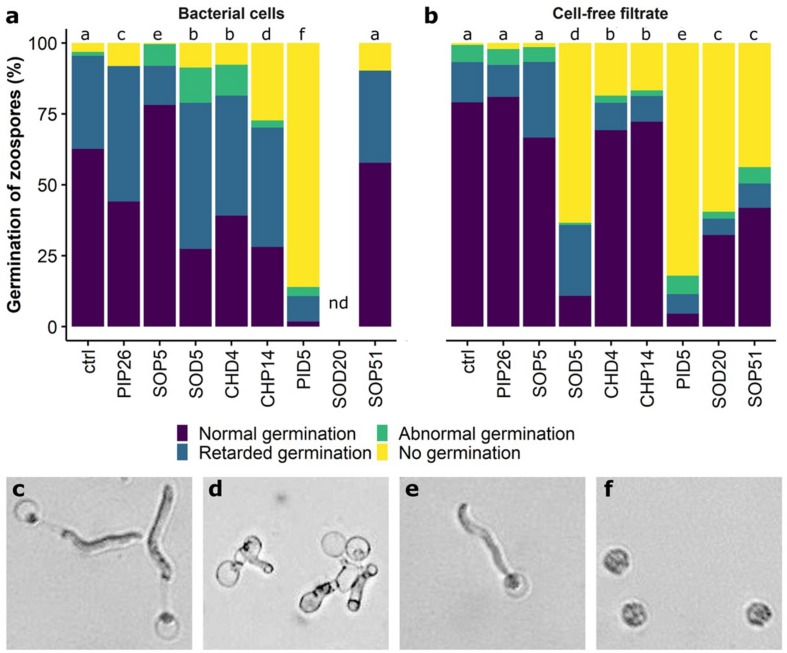
Perturbed development of germinated zoospores of *P. infestans.* Proportions of anomalies observed in bacterial cell-exposed zoospores are shown in **(a)**, those observed in cell-free filtrate-exposed zoospores are shown in **(b)**. nd, no data (the visibility on the pictures was limited due to the presence of the bacteria). Different letters indicate significantly different proportions of anomalies according to a Chi-square test with Yate’s correction for continuity (*p* < 0.05). The phenotypes observed in germinating zoospores of *P. infestans* are shown in **(c–f)**: **(c)** Normal germination; **(d)** abnormal germination; **(e)** retarded germination; **(f)** no germination. The experiment was repeated twice with similar results.

Mycelial growth of *P. infestans* upon direct exposure to the six selected *Bacillus* strains was reduced from 86.2 to 96% (Figure 2 and Supplementary Table S2). The effects of direct exposure to the same bacterial strains and their spent media on zoospore and sporangia germination (Figure 6) were more variable. While zoospore germination was slightly reduced by the presence of cells of PIP26, it was seemingly unaffected by the presence of SOP5, SOD5, CHD4, and SOP51 (Figure 6A). Although this latter strain led to complete absence of spore germination in *B. cinerea*, only PID5 and to a lesser extent CHP14 distinctly reduced the germination of zoospores of *P. infestans* (Figure 6A). Moreover, the phenotypes of the germinated zoospores appeared to be affected by all strains except SOP51 (Figure 7a). When in the presence of PIP26, SOD5, CHD4, and CHP14, the proportions of zoospores showing retarded germination (i.e., germination without appressorium formation, Figure 7e) were higher (Figure 7a). For the latter three *Bacillus* strains (SOD5, CHD4, and CHP14), the proportion of zoospores showing anomalies (Figures 7c–f) was higher than in the control. For SOP5, used as a negative control in these experiments, the reverse effect was observed (less retarded germination) but the proportion of zoospores showing anomalies was still high (Figure 7a). Zoospore germination was strikingly affected by PID5, showing only 1.75% of normal germination on average. Though for *B. cinerea* only germ tube length and not germination percentage was strongly reduced by PID5, the cells and their spent medium both seemed to specifically block the germination process of *P. infestans* zoospores (Figures 7a,b). Interestingly, when exposed to the spent medium, percentage of zoospore germination dropped significantly for SOD5, SOD20, and SOP51, whereas for CHP14, it was higher when compared to the bacterial cells (Figure 6B). However, the germination inhibition observed with bacterial cells (Figure 6A) might be underestimated since in some treatments (e.g., SOD20), very few zoospores could be found, which may be due to aggregation with bacterial cells and/or degradation of zoospores by the bacteria (Figure 6E). A closer look at the germination phenotypes revealed strong reductions in normally germinating zoospores for the spent medium of SOD5 and PID5, and a higher proportion of delayed germination for the SOD5 cell-free filtrate (Figure 7b). On the contrary, the effects of the *Bacillus* strains on sporangia germination were stronger for the bacterial cells when compared to the cell-free filtrates (Figures 6C,D). Despite an overall low germination rate of sporangia, all strains but SOP5 and SOD20 reduced sporangia germination. CHP14 and SOP51 were the strongest inhibitors of sporangia germination, as observed for *B. cinerea* spores (Figure 4A).

### Anti-oomycete Volatiles Emitted by Selected Epiphytic Grapevine Isolates

**FIGURE 8:**
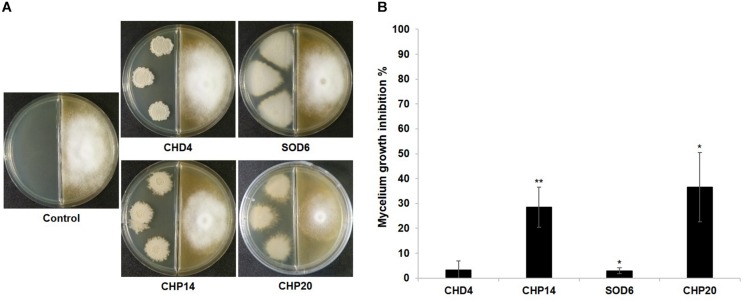
Volatile-mediated growth inhibition of *P. infestans* by selected strains. Representative pictures of dual assays with *P. infestans* non-exposed to the bacteria (Control) or exposed to four bacterial strains isolated from the grapevine microbiome are shown in **(A)**. The pictures were taken 2 weeks after *P. infestans* inoculation. The percentage of *P. infestans* mycelium inhibition induced by each strain compared to the non-exposed control that represents 100% is shown in **(B)**. Bars represent average inhibitions of 3–5 replicates with standard deviation. The percentage of inhibition was determined 2 weeks after *P. infestans* inoculation. Asterisks represent statistically significant differences in comparison to the control (*t*-test; ^∗^*p* < 0.05; ^∗∗^*p* < 0.01).

**FIGURE 9:**
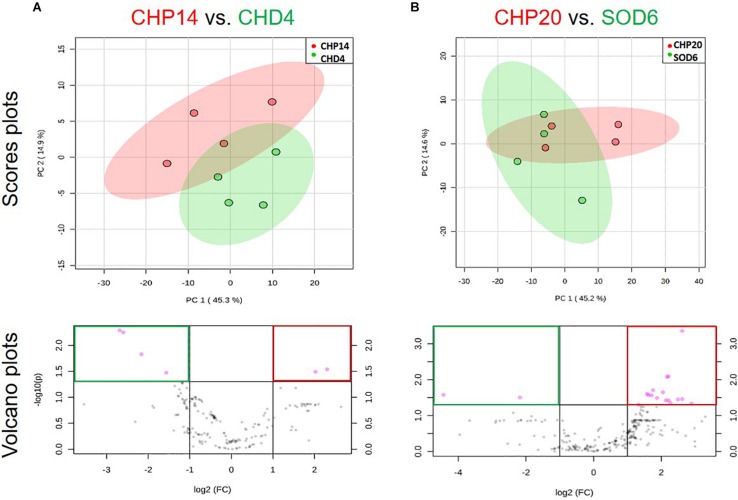
Multivariate analysis of volatiles emitted by active vs. closely related non-active strains. Two dual strain comparisons are shown: **(A)** CHP14 vs. CHD4; **(B)** CHP20 vs. SOD6. Active strains are shown in red, inactive relatives in green. Principal component analysis (PCA) score plots are shown in the upper row. These PCA plots display data variance over two principle axes, with explanatory percentages shown in brackets. Volcano plots displaying significance over fold-change are shown in the lower row. Mass features varying with high significance (*p* < 0.05) and fold-change (>2) are displayed in pink and framed red for features associated with active strains vs. green for those associated with inactive strains.

Using a two-compartment Petri dish assay in our screening, we observed significant reduction of mycelial growth when the oomycete pathogen *P. infestans* was exposed to volatiles from three epiphytic strains isolated from the Chasselas cultivar (Figure 2). In contrast to the phylogenetically closely related *Botrytis-*inhibiting *Bacillus* isolates described above, these three strains (CHP14, CHP20, and CHP33) belonged to three different subgroups: *B. subtilis* for CHP14, *B. cereus* for CHP20 and *Frigoribacterium* for CHP33 (Figure 1). We expected their volatile blends to differ too strongly to allow identification of common volatiles involved in anti-oomycete activity. In order to identify the volatiles responsible for the mycelial growth reduction, we therefore looked for closely related strains that lacked such volatile-mediated activity. While no close relative could be found for CHP33, which is why we did not include this strain in the analysis, we selected CHD4 as “inactive counterpart” for CHP14, and SOD6 for CHP20. The differing activity of these four strains is shown with representative pictures in Figure 8. To compare their overall volatile profiles, we performed a principal component analysis (PCA) on both couples of active vs. inactive strains (Figure 9). As shown in the PCA score plots, there was – as expected for closely related strains - partial overlap between the volatilomes of the active vs. non-active strains, but multivariate analysis revealed features (mass ions corresponding to specific compounds) that were significantly different between the active and the inactive strain of each couple (in pink in the Volcano plots). The features that are of interest in our case are those enriched in or specific to active strains and can be found in the top right of the plots (Figure 9). The corresponding molecules are listed in Table 2. Only one of the two compounds that were significantly more abundant in the volatilome of CHP14 compared to CHD4 could be identified with confidence: the tryptophan derivative L-dihydroxanthurenic acid (Figure 9A and Table 2). When comparing the volatiles emitted by the active CHP20 with those emitted by its closely related yet less active counterpart SOD6, a stronger overlap was observed in the PCA scores plot for this couple than for the CHP14/CHD4 couple (Figure 9). Nevertheless, three compounds were significantly enriched in CHP20: trimethylpyrazine, dihydrochalcone, and the L-dihydroxanthurenic acid already detected in CHP14 (Figure 9B and Table 2).

**TABLE 2:** Volatile compounds enriched in active strains, as revealed by multivariate analysis on log transformed, normalized GC/MS data using MetaboAnalyst 3.0.

**RT**	**m/z**	**Name**	**CAS**	**Match**	**Strain**
19.17	43.05 162.05 58.05 99.05 42.05.	L-Dihydroxanthurenic acid	4886-42-4	76.45	CHP14
*28.89*	*55.05 83.15 69.05 43.05 41.05.*	*Unknown*		*8.23*	*CHP14*
10.36	122.05 42.05 44.05 39.05 40.05.	Trimethylpyrazine	14667-55-1	84.65	CHP20
19.17	43.05 162.05 99.05 58.15 42.15.	L-Dihydroxanthurenic acid	4886-42-4	69.64	CHP20
*28.89*	*55.05 83.05 69.05 43.05 57.05.*	*Unknown*		*8.35*	*CHP20*
*29.83*	*91.05 208.15 104.05 130.05 115.05.*	*Unknown*		*37.92*	*CHP20*
32.61	105.05 210.05 77.05 91.05 51.05.	Dihydrochalcone	1083-30-3	82.75	CHP20
*44.65*	*91.05 207.15 129.05 206.15 105.05.*	*Unknown*		*50.09*	*CHP20*
*46.79*	*91.05 129.05 312.15 180.05 207.05.*	*Unknown*		*58.22*	*CHP20*

*Compounds listed were significantly more abundant (*p* < 0.05) by a factor of at least two in active than in non-active strains. Identification of the enriched compounds was carried out based on the NIST17 database using the OpenChrom software. RT, retention time; m/z, mass/charge; Match, probability of correct compound identification. Lines in italic represent compounds identified with low match quality and therefore classified as “unknown.”*

## Discussion

### Diversity of Cultivable Bacteria in the Leaves of Three Grapevine Cultivars

The major aim of this study was to isolate and identify new potential candidates for biological control of fungal and oomycete grapevine pathogens rather than to provide a comprehensive view of the leaf microbiota. This is why we focused on a cultivation-based approach to compare the endophytic and epiphytic bacterial communities present on three different grapevine cultivars grown on the same organically managed experimental vineyard in Prangins (Switzerland) (Vionnet et al., 2018). One striking feature in our survey was the overrepresentation of *Bacillus* and *Staphylococcus* species in all three cultivars, and especially among endophytic communities (Figure 1). Bacteria belonging to these genera are readily cultivable on standard microbiology media such as the PCA (Plate Count Agar) used in our study (Vionnet et al., 2018), which might partially explain their dominance. However, members of both genera have also been identified in cultivation-independent surveys of phyllosphere grapevine microbiota (Campisano et al., 2014; Perazzolli et al., 2014; Yousaf et al., 2014; Singh et al., 2018). Vineyard management was shown to greatly influence not only the soil and rhizosphere (Calleja-Cervantes et al., 2015; Vega-Avila et al., 2015) but also the phyllosphere microbiota (Campisano et al., 2014). Notably, higher proportions of *Staphylococcus* and other commensal and opportunistic pathogens associated with animals and humans were observed in the endosphere of grapevine from organically managed vineyards (Campisano et al., 2014; Yousaf et al., 2014), which might be linked to the use of organic manure rather than mineral fertilizer. Such organic amendments are applied to the soil, but since above-ground microbial communities in grapevine have been shown to share a substantial proportion of their taxa with the soil (Zarraonaindia et al., 2015), it seems likely that microbes contained in organic manure might also colonize above-ground plant parts. Similarly to our study, few recent reports focusing on cultivable bacteria from grapevine leaves also identified *Bacillus* isolates as major components of endophytic communities (Baldan et al., 2014; Andreolli et al., 2016). In addition, they detected other genera such as *Methylobacterium* and *Pantoea* (Andreolli et al., 2016) or *Staphylococcus, Paenibacillus, Microbacterium, Micrococcus*, and *Variovorax* (Baldan et al., 2014), which were also isolated in the present study, either among endophytes or epiphytes (Figure 1). This relatively strong overlap at genus level is surprising when considering that these studies investigated different cultivars, i.e., Corvina (Andreolli et al., 2016) and Glera (Baldan et al., 2014), and that plant genotype is known to play a major role in shaping rhizosphere and phyllosphere microbiotas (Cardinale et al., 2015; Wagner et al., 2016; Berlanas et al., 2019). In our study, differences were observed between the three cultivars, especially among epiphytes (Figure 1): higher cultivable diversity was found in Pinot Noir than in Chasselas and Solaris, which was associated with a lower proportion of *Bacillus* strains than in the two other cultivars. Among endophytes, all three cultivars shared a dominance of the genera *Bacillus* and *Staphylococcus*, although the proportions between these two varied between the cultivars (Figure 1 and Supplementary Table S1). Apart from these rather minor differences between cultivars, the main differences in cultivable diversity occurred between epiphytes and endophytes: while only few genera were represented among endophytes, the leaf surface contained higher diversity, and notably a higher proportion of Gram-negative bacteria, such as the two well-described leaf inhabitants *Sphingomonas* and *Methylobacterium* (Müller et al., 2016; Compant et al., 2019). Lower diversity among endophytes than epiphytes is expected (Bulgarelli et al., 2013) and can be linked to a stronger selection pressure by the plant and to the necessity for endophytes to be able to overcome additional barriers, such as the endodermis in roots or the cuticle in leaves. Moreover, the stronger overlap between the three cultivars among endophytes than among epiphytes (Figure 1) might relate to tight association between endophytes and their host. In contrast to endophytic communities, epiphytic communities might harbor both highly adapted strains selected for their ability to withstand harsh abiotic conditions (e.g., UV, desiccation), and “transient passengers” brought by wind and rain but lacking the specific equipment necessary to establish stable populations in the phyllosphere. Current isolation procedures do not allow distinguishing between these two types of epiphytes and identifying plant-beneficial strains in this particular compartment thus does not warrant their long-term survival in the plant phyllosphere. In contrast, endophytes may represent a more promising pool of isolates to search for host-adapted microbes with plant-beneficial functions, such as health protection through inhibition of plant pathogens (Brader et al., 2014; Hardoim et al., 2015).

### Antagonistic Activity of Grapevine Leaf Isolates Against *Botrytis cinerea*

When comparing the antagonistic potential of epiphytic vs. endophytic isolates on two phytopathogens, we observed that a high proportion of endophytes was able to inhibit mycelial growth of both pathogens in full plate assays, while this ability was less widespread among epiphytes (Figure 2 and Supplementary Table S2). As discussed above, this might be due to lesser selection pressure on plant surface- than on inner tissue-colonizing bacteria, although further studies on a more extensive strain collection would be needed to confirm this hypothesis. In such full plate assays where both diffusible and volatile compounds can be exchanged between the two partners, only seven strains (three epiphytes and four endophytes) were able to strongly inhibit *B. cinerea* mycelial growth (>50%, red color in Figures 2, 3). From these seven strains, six belonged to the genus *Bacillus* (Figure 1). *Botrytis cinerea* is one of the major fungal pathogens worldwide, causing great pre- and post-harvest losses in a wide range of agronomically relevant crops (Dean et al., 2012). One key element promoting fast disease dispersion of this pathogen is the massive production of conidia that are easily spread by wind (Williamson et al., 2007). An efficient biocontrol agent should therefore not only inhibit mycelial growth but also prevent spore formation or germination. We thus tested whether the six *Bacillus* grapevine isolates showing similar and high inhibition of *B. cinerea* mycelial growth would also inhibit spore germination. This was indeed the case for five of them, while the sixth (SOD5), which clustered in a different clade closely related to *B. licheniformis* (Figure 1), did not interfere at all with spore germination (Figures 4, 5). From the five remaining strains, different phenotypes were observed, some strains (e.g., CHD4) showing no effect on germination rate but leading to smaller germ tubes, others strongly affecting both germination rate and germ tube length (e.g., CHP14 and SOP51). Specific anomalies in germ tube development were observed for some strains or groups of strains: CHD4 filtrate led to a high proportion of hyphal swelling at the germ tube tip, and the three closely related PID5, SOD20 and SOP51 induced similar disturbances when applied as cell-free filtrates, while they differed in their effects when applied as cell suspensions (Figure 5). This suggests that the spent medium of these three strains contained the same (or similar) active compounds. While we did not determine their identity in this study, one likely candidate class of compounds that could be responsible for the observed effects is the lipopeptides. Indeed, *Bacillus* strains are well known for their ability to produce a wide range of such surface-active molecules with broad effects on phytopathogens (Ongena and Jacques, 2008; Liu et al., 2014), some of which were shown to inhibit *B. cinerea* development (Touré et al., 2004; Haggag, 2008; Zhang et al., 2013). Beyond lipopeptides, other antimicrobial compounds produced by *Bacillus* strains, such as polyketides (e.g., macrolides), or lytic enzymes (e.g., chitinases), could also be involved in the observed effects (Caulier et al., 2019). Reports of volatile-mediated activity of *Bacillus* strains on *B. cinerea* also exist (Chen et al., 2008; Liu et al., 2008; Zhang et al., 2013), but this pathogen was not inhibited in its growth in our volatile assays, in contrast to the oomycete *P. infestans*.

### Antagonistic Activity of Grapevine Leaf Isolates Against *Phytophthora infestans*

Downy mildew caused by the oomycete *Plasmopara viticola* is one of the most important diseases threatening grapevine health (Gessler et al., 2011; Kamoun et al., 2015). This pathogen is an obligate parasite, which makes *in vitro* assays such as the screen of grapevine isolates for antagonistic activity very challenging. This is why we have selected a closely related pathogen, the oomycete *P. infestans* as a surrogate for the early selection of strains with anti-oomycete activity. Although both pathogens might react differently to biological control agents, earlier work on *Lysobacter* strains revealed their efficacy against both pathogens (Puopolo et al., 2014a, b), which raises hope that some of the strains identified as inhibitors of *P. infestans* might also inhibit *P. viticola.* Our screening revealed that in contrast to *B. cinerea*, which was inhibited only by few *Bacillus* strains, *P. infestans* mycelial growth was significantly reduced by a number of grapevine isolates affiliated with different genera (*Bacillus*, *Staphylococcus* and *Micrococcus* among Gram-positive and *Variovorax, Pantoea, Herbaspirillum* and *Erwinia* among Gram-negative) (Figure 2). This suggests higher sensitivity of the oomycete to the diffusible metabolites produced by the bacteria during full plate confrontation. A high proportion of endophytes severely inhibited *P. infestans* mycelial growth, and they mostly belonged to the *Bacillus* genus. As discussed above for *B. cinerea*, lipopeptides might be involved in this strong growth inhibition, as susceptibility of this pathogen (and of *P. viticola*) to different lipopeptides has been reported (Stanghellini and Miller, 1997; Tran et al., 2007; Zachow et al., 2015). In addition, small cyclic peptides such as those produced by *Lysobacter capsici* might also play a role in this inhibition (Nishanth Kumar et al., 2012; Puopolo et al., 2014a). Both types of compounds (lipopeptides and small cyclic peptides) should be contained in the cell-free filtrates and indeed, the filtrates of the three most active strains against *B. cinerea* (PID5, SOP21 and SOP51) also strongly perturbed *P. infestans* zoospore germination, indicating broad range activity of the secreted molecules. Beyond *Bacillus* strains and in view of the broad phylogenetic distribution of strains that reduced *P. infestans* mycelial growth through diffusible compounds, a similarly broad chemical diversity of responsible molecules (Stringlis et al., 2018) might underlie the growth inhibition and identifying these molecules shall be the scope of future studies. In contrast to diffusible compound-mediated growth inhibition, reduction of *P. infestans* mycelial growth by the emission of volatile compounds seemed largely restricted to *Bacillus* strains (with the exception of one *Frigoribacterium*, CHP33), and specifically to those isolated as epiphytes from the Chasselas cultivar (Figure 2). Although volatiles emitted on nutrient-rich media such as LB might only very partially resemble those emitted on leaf surfaces (Blom et al., 2011), they still provide an idea of the metabolic potential of strains showing volatile-mediated inhibition of pathogens *in vitro.* When comparing the volatilomes of these active isolates to those of phylogenetically closely related, but non-active isolates, we identified few volatiles enriched (or only detected) in active strains. These compounds, which might therefore be involved in the volatile-mediated growth inhibition of the oomycete pathogen (Figure 9 and Table 2), included trimethylpyrazine, which is known to have antifungal activity (Méndez-Bravo et al., 2018). Interestingly, one compound appeared as enriched in both active strains compared with their inactive counterparts: L-dihydroxanthurenic acid. Very little is known about this molecule, which appears to be produced in insects by a kynurenine transaminase (Dolores Real and Ferré, 1991). Given the molecular structure of this tryptophan derivative, one might indeed speculate that it is an intermediate in the kynurenine pathway, which has been implicated in the biosynthesis of (non-volatile) antimicrobial and cell-cell communication signals such as quinolones (Gross and Loper, 2009) or the siderophore thioquinolobactin involved in anti-oomycete activity (Matthijs et al., 2007). Moreover, a recent study identified a genomic locus in a *Pseudomonas* strain involved in anti-oomycete activity, which contained several genes encoding different steps of the kynurenine pathway (Wagner et al., 2018). Although the biosynthesis and biological activity of L-dihydroxanthurenic acid is so far unknown, this newly detected volatile might be an interesting candidate for further studies of its potential implication in the inhibition of oomycetes such as *P. infestans* or *P. viticola.*

### Harnessing the Potential of Grapevine Endophytes and Epiphytes for Sustainable Disease Control

Overall, our results show that grapevine leaves are a rich source of potential biocontrol agents of fungal and oomycete pathogens. There was no striking difference in the relative abundance of antagonistic strains between the different cultivars, although emitters of anti-oomycete volatiles were all isolated from the disease-sensitive Chasselas cultivar. More comprehensive surveys on larger pools of sensitive vs. resistant cultivars would be needed to draw conclusions about the role of the phyllosphere microbiome in disease resistance, which was not the aim of the present study. However, it seems from our results that leaf isolates producing diffusible compounds inhibiting pathogen growth were more frequently retrieved in the endophytic communities, while all emitters of anti-oomycete volatiles belonged to epiphytic communities. Indeed, volatile compounds are likely more efficient in fending off disease-causing agents when emitted on the surface of the plants, in contact with air, which allows their dispersion to places where spores of pathogens might land. In contrast, diffusible substances and contact-mediated inhibition of penetrating pathogens might constitute a further line of defense provided by bacteria living in the leaf endosphere. Accordingly, providing grapevine with particularly health-protective endophytes (e.g., by soil drenching) at the beginning of the season, could be combined with later leaf spraying treatments with epiphytic emitters of pathogen-inhibiting volatiles. Together with the use of disease-resistant cultivars and continuous monitoring of disease pressure to allow timely treatments, such application of biological agents adapted to their host plant might contribute to efficient disease control strategies of lower environmental impact than the traditionally used synthetic fungicides.

## Data Availability Statement

The raw data supporting the conclusions of this manuscript will be made available by the authors, without undue reservation, to any qualified researcher.

## Author Contributions

LW, FL’H, and MD designed the research. SB, MZ, FL’H, ET, MD, AA, and AD performed the experiments. SB, MZ, FL’H, ET, AA, and MD analyzed the data. LW, FL’H, SB, MZ, MD, and AA wrote the manuscript with help from ET.

## Conflict of Interest

The authors declare that the research was conducted in the absence of any commercial or financial relationships that could be construed as a potential conflict of interest.

## Footnotes

1 www.fisher.co.uk

2 www.carlroth.com

3 www.junkfood.ch

4 https://blast.ncbi.nlm.nih.gov/Blast.cgi
