# Supplementary figures and images for: Endophytes and Epiphytes From the Grapevine Leaf Microbiome as Potential Biocontrol Agents Against Phytopathogens
Source: Front Microbiol. 2019 Nov 29;10:2726. doi: 10.3389/fmicb.2019.02726 (PMC6895011; doi:10.3389/fmicb.2019.02726)

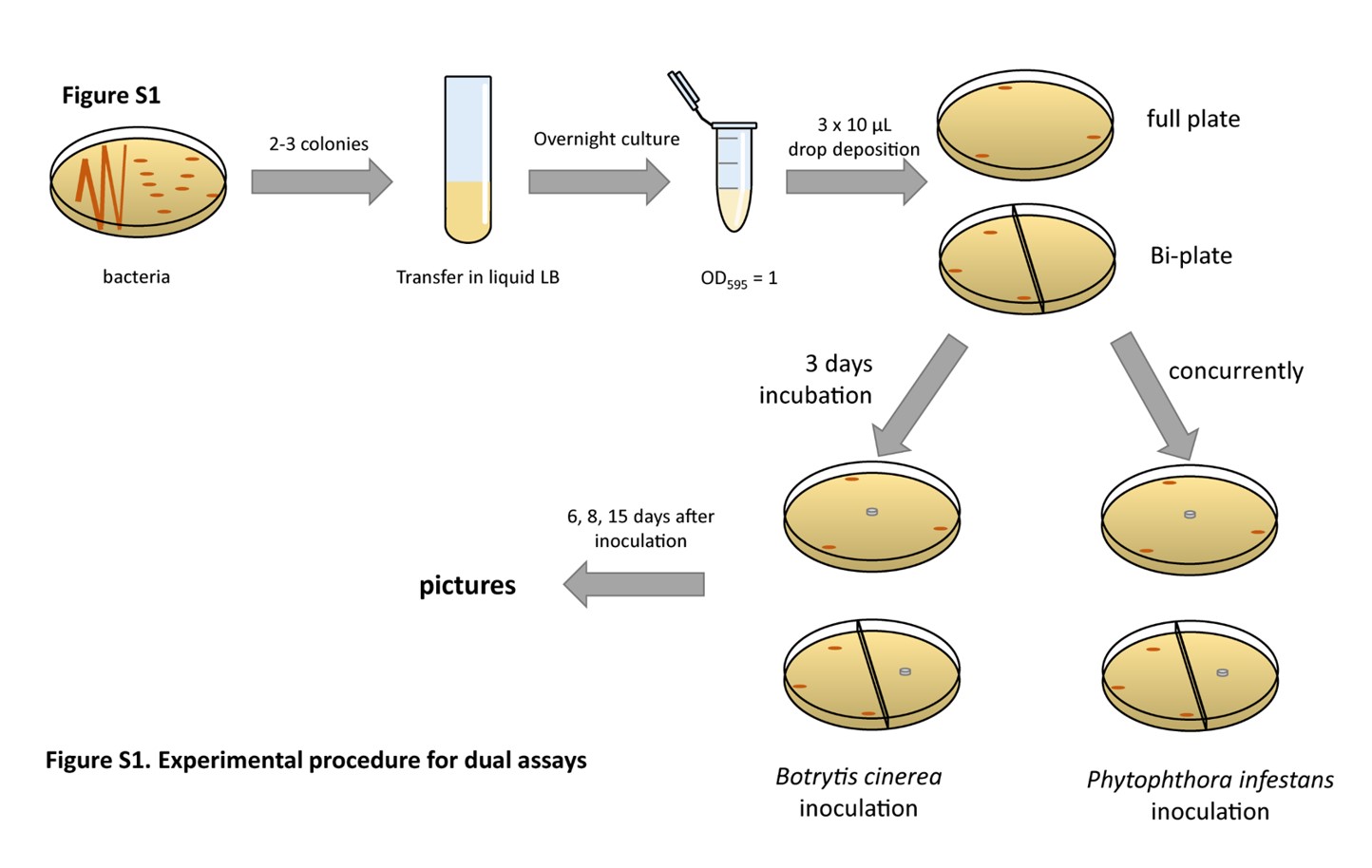

Supplement: Supplementary file 3 [file Image_1.jpg]

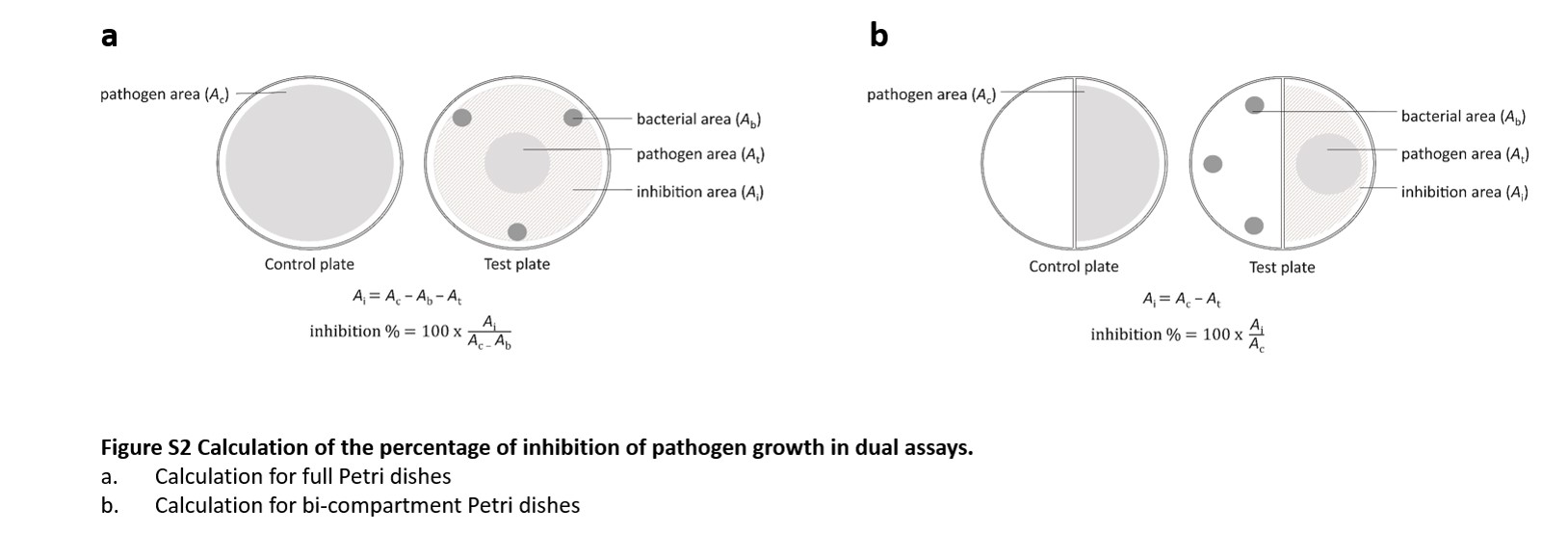

Supplement: Supplementary file 4 [file Image_2.jpg]

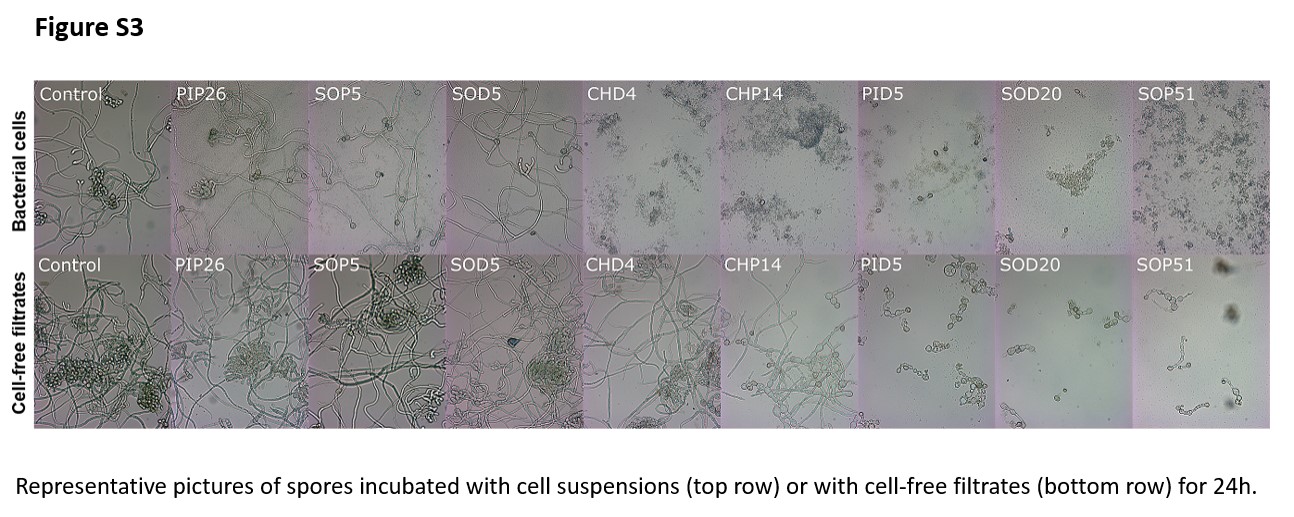

Supplement: Supplementary file 5 [file Image_3.jpg]
